# A simple framework for maximizing camera trap detections using experimental trials

**DOI:** 10.1007/s10661-023-11945-9

**Published:** 2023-10-27

**Authors:** Philip D. DeWitt, Amy G. Cocksedge

**Affiliations:** Science and Research Branch, Ministry of Natural Resources and Forestry, 300 Water Street, Peterborough, Ontario K9J 3C7 Canada

**Keywords:** Ecology, Detection probability, Distance sampling, Passive infrared sensor, Remote sensing, Wildlife

## Abstract

Camera trap data are biased when an animal passes through a camera’s field of view but is not recorded. Cameras that operate using passive infrared sensors rely on their ability to detect thermal energy from the surface of an object. Optimal camera deployment consequently depends on the relationship between a sensor array and an animal. Here, we describe a general, experimental approach to evaluate detection errors that arise from the interaction between cameras and animals. We adapted distance sampling models and estimated the combined effects of distance, camera model, lens height, and vertical angle on the probability of detecting three different body sizes representing mammals that inhabit temperate, boreal, and arctic ecosystems. Detection probabilities were best explained by a half-normal-logistic mixture and were influenced by all experimental covariates. Detection monotonically declined when proxies were ≥6 m from the camera; however, models show that body size and camera model mediated the effect of distance on detection. Although not a focus of our study, we found that unmodeled heterogeneity arising from solar position has the potential to bias inferences where animal movements vary over time. Understanding heterogeneous detection probabilities is valuable when designing and analyzing camera trap studies. We provide a general experimental and analytical framework that ecologists, citizen scientists, and others can use and adapt to optimize camera protocols for various wildlife species and communities. Applying our framework can help ecologists assess trade-offs that arise from interactions among distance, cameras, and body sizes before committing resources to field data collection.

**Supplementary Information:**

The online version contains supplementary material available at 10.1007/s10661-023-11945-9.

## Introduction

Camera traps are widely used to collect wildlife data that can inform conservation and management decisions at local, ecosystem, and landscape levels (Keim et al., 2021; Swanson et al., 2015; Visscher et al., 2017, 2023). They have become a standard method of monitoring wildlife across varying spatial and temporal scales (Meek et al., 2014; Steenweg et al., 2017) because of their ability to efficiently record multiple species using non-invasive, autonomous methods. Camera trap data can be used to assess wildlife distribution, diversity, composition, abundance, density, population trends, and behaviour provided they are deployed following a rigorous sampling design (Burton et al., 2015; McIntyre et al., 2020; Rovero et al., 2013).

Like many monitoring approaches, camera traps can result in false negatives where an animal is present but not recorded. The detection process consists of a series of conditional probabilities (Hofmeester et al., 2019) that can be formulated as (Kays et al., 2021):

$${p}_i=1-{\left[1-\left({r}_e \cdot {r}_t \cdot {r}_p\right)\right]}^{N_i}$$

where the probability of observing an animal (*p*_*i*_) in time interval *i* is a product of three parameters and the number of visits during that interval (*N*_*i*_). The parameters relate to the conditional probabilities of an animal encountering the camera given it is within the sample unit (*r*_*e*_), triggering the camera once it has encountered the camera (*r*_*t*_), and the camera capturing a usable image of the animal (*r*_*p*_). These processes are sometimes combined into a single probability estimate (Burton et al., 2015); however, understanding each component is valuable when inferring ecological phenomena at broad spatial scales (Kays et al., 2021) or using camera data to obtain information about the encounter process (Findlay et al., 2020; Moeller et al., 2018; Nakashima et al., 2018).

The probability of detecting an animal passing through a camera’s field of view (*r*_*t*_) arises from the interaction between the camera and the animal. Most camera traps use passive infrared (PIR) sensors that detect thermal energy from the surface of an object. These sensors are triggered when a difference in heat moves between sensors (Meek et al., 2014; Welbourne et al., 2016). Cameras are therefore more effective at detecting animals with large surface areas relative to the sensor array. For example, the probability of detecting animals declines with both body size and distance (Heiniger & Gillespie, 2018; Howe et al., 2017; Jacobs & Ausband, 2018; Mason et al., 2022; Rowcliffe et al., 2011) because their heat signatures appear smaller. Similarly, camera type, height, angle, and target species can impact *r*_*t*_ by changing the relationship between a sensor array and an animal (Apps & McNutt, 2018; Kelly & Holub, 2008; Welbourne et al., 2016).

Given the growing demand for data to support the conservation and management of multiple species (i.e. biodiversity), quantifying detection can help ecologists make a priori decisions about camera trap deployments and the subsequent analysis and interpretation of data. Here, we describe a general, experimental approach that extends distance models to evaluate detection errors that occur when an animal passes through a camera’s field of view but is not recorded (*r*_*t*_). Using two camera models commonly used in wildlife research (Reconyx HP2X and PC900), we first calculated each model’s horizontal field of view. We then estimated the effect of distance, camera lens height, model, and vertical angle on the probability of detecting three different body sizes representing mammals ranging in size from red fox (*Vulpes vulpes*) to moose (*Alces alces*). Our goal was to develop a general, experimental, and analytical framework that ecologists, citizen scientists, and others can use, and adapt, to maximize detection probabilities (*r*_*t*_) for a given wildlife species or community.

## Materials and methods

### Data collection

We conducted experiments in a flat, grassy field in Ontario, Canada (44.36° N, 78.74° W). The treeless field covered 2500 m^2^ so it was unlikely to result in false negatives resulting from topographic variation, or false positives resulting from movement of background surface temperatures (Welbourne et al., 2016). All experiments were completed using a standardized plot. Three cameras were mounted to a single pole with the bottom of the cameras located 80, 110, and 140 cm above ground level, corresponding to lens heights of 86, 116, and 146 cm respectively. Cameras cannot detect animals when covered by snow, and these heights represent a range of conditions that accommodate mid-winter snow depths throughout many temperate and boreal ecosystems. We placed stakes 2, 4, 6, 8, 10, 12, and 15 m in front of the cameras and marked the maximum extent of the plot by placing stakes at 1 m intervals perpendicular to the 15 m stake (Appendix 1, Figs. 4 and 5). We placed a 160-cm-tall snow measurement gauge in front of the camera array and positioned each camera so the gauge appeared in the centre of the field of view, with the top of the gauge in the top 25% of the image. Finally, we measured the horizontal field of view and vertical angle of the cameras used in the experiment (Appendix 2).

We conducted a series of experimental detection trials using proxies with similar shoulder heights of furbearers and game species that inhabit boreal and temperate ecosystems. The medium proxy was a person crawling with a height of 30–50 cm, representing animals such as red fox and lynx (*Lynx* spp.). The large proxy was a person walking hunched over with a height of 80–110 cm, representing grey wolf (*Canis lupus*), wild boar (*Sus scrofa*), American black bear (*Ursus americanus*), white-tailed deer (*Odocoileus virginianus*), fallow deer (*Damas dama*), roe deer (*Capreolus* spp.), caribou and reindeer (*Rangifer tarandus*), and red deer (*Cervus elaphus*). Finally, the large ungulate proxy was two people walking side-by-side with a height of 160–195 cm, representing wapiti (*Cervus canadensis*), bison (*Bison* spp.), and moose.

**Fig. 1:**
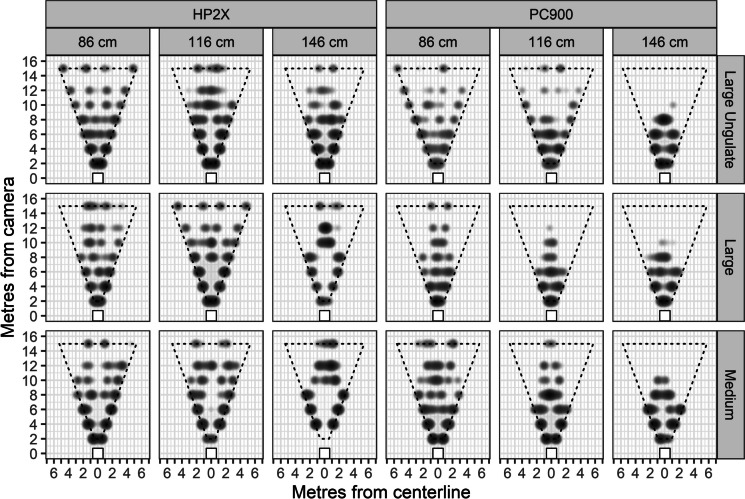
The kernel density (bandwidth = 1.5) of detections for each camera model, lens height, and proxy body size combination. Darker clouds represent a higher intensity of observations at that location than lighter clouds. The dotted lines are the estimated fields of view for the HP2X and PC900 camera models

We programmed cameras to capture 5 images when triggered, wait 0 s between triggers, and rearm. We conducted ten trials for every combination of proxy (medium, large, and large ungulate), camera model (PC900, HP2X), lens height (86, 116, and 146 cm), snow measurement gauge distance (hereafter ‘aiming distance’; 5, 10, and 15 m), and distance (2, 4, 6, 8, 10, 12, and 15 m) resulting in a balanced dataset consisting of 3780 trials. Each trial was timed and conducted by moving perpendicular to the camera array. We waited at least 15 s between trials to ensure each trial was independent and alternated the direction of movement so that proxies entered the field of view from both directions. We manually inspected every image, determined whether each trial resulted in a detection, and mapped the location where the proxy was first detected by comparing its position to the stakes in the photo (Fig. 1).

### Detection modelling

We estimated the probability of detecting proxies in two steps. First, we adapted distance sampling models to a binomial response and fit empirical detection functions based on the exponential, half-normal, and hazard distributions:

$$p(y)=\exp \left(-y/\alpha \right)$$

$$p(y)=\exp \left(-{y}^2/\left(2\ast {\alpha}^2\right)\right)$$

$$p(y)=1-\exp \left(-{\left(y/a\right)}^{-\gamma}\right)$$

where *p*(*y*) is the detection probability at *y* distance, *α* is the scale parameter, and *γ* is the shape parameter. We considered candidate models consisting of standard functions where detection declines monotonically with distance, as well as modified functions that include a logistic mixture model that account for animals passing beneath the camera (Rowcliffe et al., 2011). We fit distance models using maximum likelihood methods in the R bbmle package (Bolker and R Development Core Team, 2022) and selected the most parsimonious model using AIC.

Second, we estimated the mean probability of detection in relation to experimental covariates (i.e. proxy body size, proxy distance, camera model, lens height, aiming distance, vertical angle), as well as proxy speed, sun altitude above the horizon (radians), and sun azimuth (radians) while conditioning parameter estimates on the most parsimonious detection function from the first step. Camera angles were correlated with lens height (Spearman’s *ρ* = 0.71, *p* < 0.001) and aiming distance (*ρ* = 0.47, *p* < 0.001) so we fit one suite of models considering vertical angle and a second suite considering lens height and aiming distance. We also considered statistical interactions between body size and distance, based on the expectation that larger animals are more easily detected than smaller animals when further away, and between camera model and distance. We fit global models and used backwards stepwise selection until only significant covariates and interactions remained in each model. Binomial regression models were estimated by using generalized linear models with a logit link function in R 4.2.1 (R Core Team, 2022). We used the receiver-operating characteristic to evaluate the final models’ ability to correctly classify true detections (sensitivity) and misses (specificity) using the pROC package (Robin et al., 2011).

## Results

Detection probabilities were best explained by a half-normal-logistic mixture that allows lower probabilities near the camera (Table 1). All experimental factors influenced detection in both regression models. Confidence intervals around shared parameters overlapped, with most intervals overlapping the mean estimate from the complementary model (Appendix 1, Fig. 6), implying that parameters were robust to including camera angle or lens height and aiming distance. Both models had AUCs ≥0.920 (Appendix 1, Figs. 7 and 8) which is often considered to have superior discrimination (Hosmer et al., 2013).

**Table 1:** Model selection results of empirical detection functions based on the exponential, half-normal, and hazard distributions, where K is the number of estimated parameters, LL the log-likelihood, AIC the Akaike Information Criterion, and ΔAIC the difference compared to the most parsimonious model

Model	K	LL	AIC	ΔAIC
Half-normal (logistic mixture)	3	−1654.6	3315.3	0
Half-normal	1	−1934.0	3870.1	554.8
Exponential	1	−1973.1	3948.2	633.0
Exponential (logistic mixture)	3	−1973.1	3952.2	637.0
Hazard	2	−2014.3	4032.6	717.4
Hazard (logistic mixture)	4	−2014.3	4036.6	721.4

**Fig. 2:**
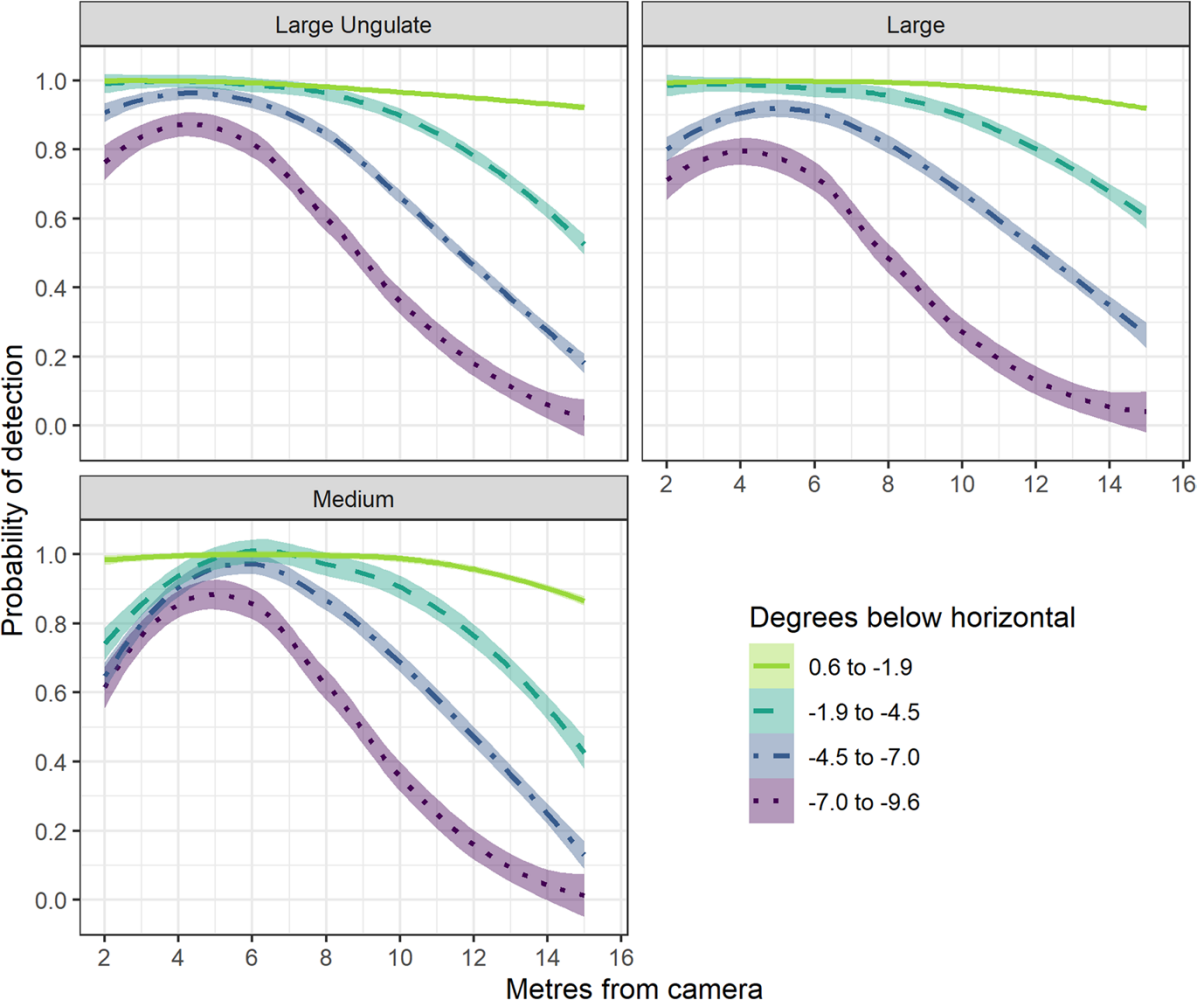
Estimated mean probabilities of detecting a proxy based on its body size, distance from the camera, and vertical angle of the camera relative to the ground surface. The estimated means and standard errors are shown in lines and shading, respectively

**Fig. 3:**
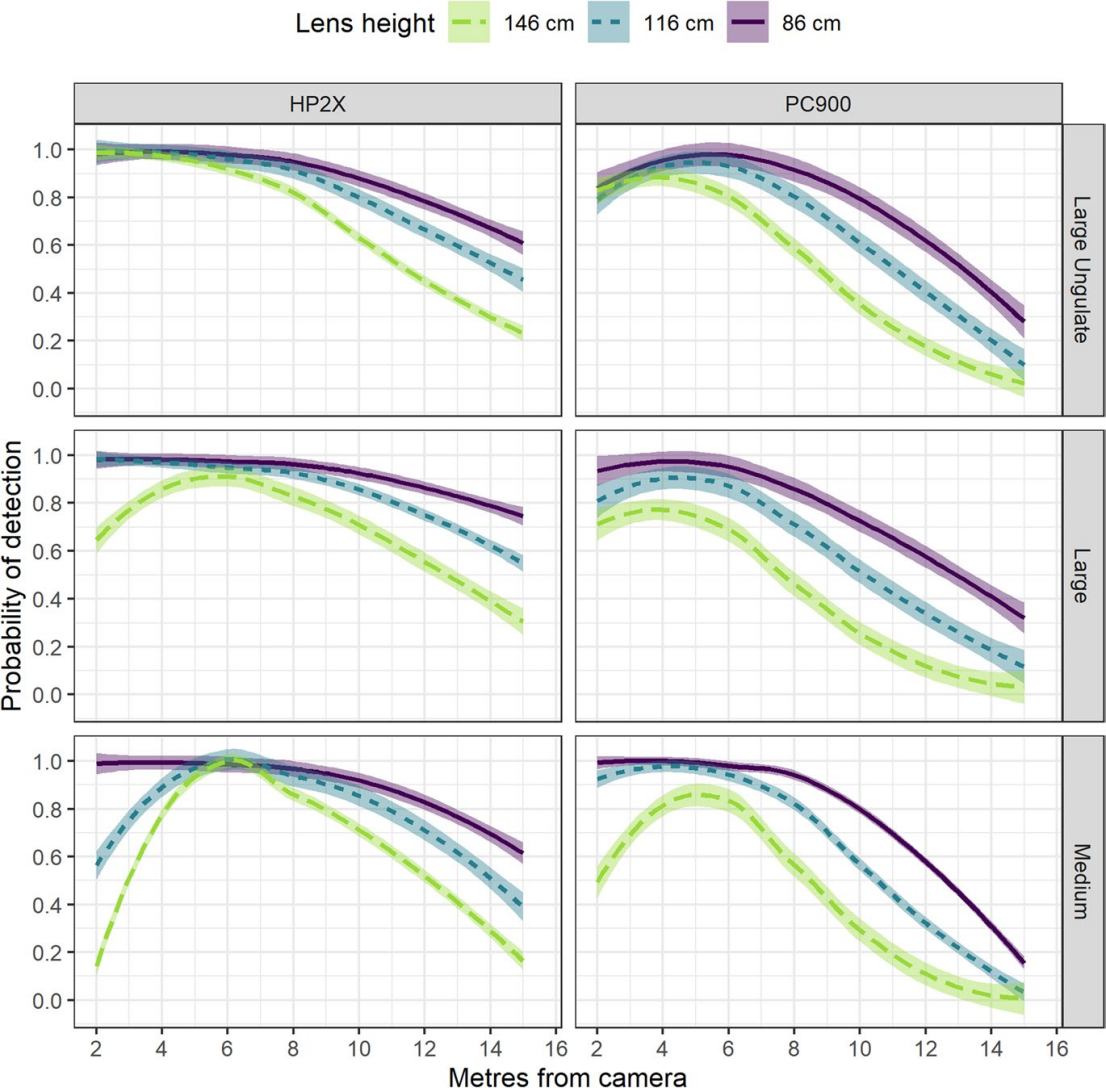
Estimated marginal probabilities of detecting a proxy based on camera model, body size, distance from the camera, and lens height. The estimated means and standard errors are shown in lines and shading, respectively

Detection monotonically declined when proxies were ≥6 m from the camera; however, our regression models show that body size and camera model mediated the effect of distance on detection (Figs. 2 and 3). The two larger proxies had similar detection probabilities across the range of experimental distances (Tables 2 and 3; *p* > 0.103). Although the medium-sized proxy had significantly lower probabilities of being detected, the magnitude of the effect varied depending on its distance from the camera (*p* < 0.001). Marginal effects show the probability of detecting the medium-sized proxy was consistent with larger proxies when located 4–12 m in front of the camera but was lower when located 2 m (lower by 0.17–0.24) or 15 m in front of the camera (lower by 0.1–0.10).

**Table 2:** Parameter estimates and standard errors in the selected binomial regression model including vertical camera angle. The model estimates the probability of detecting a medium-sized proxy and larger 2 to 15 m in front of a camera. Distance model refers to the fitted half-normal-logistic mixture model. Body size includes large ungulates (intercept term), large, and medium. Camera model includes HP2X (intercept term) and PC900. Statistical interactions are denoted with a colon

Covariates	Parameter estimate	Standard error	*Z* value	Pr(|>*z*|)
Intercept	6.803	0.854	7.969	<0.001
Vertical angle (degrees)	0.565	0.030	18.744	<0.001
Body size (large)	−0.793	0.486	−1.633	0.103
Body size (medium)	−6.262	0.685	−9.147	<0.001
Camera model (PC900)	−2.300	0.397	−5.785	<0.001
Distance model	6.281	0.646	9.726	<0.001
Speed (metres per second)	−4.970	0.945	−10.047	<0.001
Sun azimuth	−0.596	0.096	−6.238	<0.001
Body size (large) : distance model	0.491	0.729	0.674	0.501
Body size (medium) : distance model	4.080	0.783	5.210	<0.001
Camera model (PC900) : distance model	2.870	0.622	4.616	<0.001

**Table 3:** Parameter estimates and standard errors in the selected binomial regression model including lens height and aiming distance. The model estimates the probability of detecting a proxy and larger 2 to 15 m in front of a camera. Distance model refers to the fitted half-normal-logistic mixture model. Body size includes large ungulates (intercept term), large, and medium. Camera model includes HP2X (intercept term) and PC900. Statistical interactions are denoted with a colon

Covariates	Parameter estimate	Standard error	*Z* value	Pr(|>*z*|)
Intercept	10.058	0.997	10.084	<0.001
Lens height (cm)	−0.019	0.002	−8.605	<0.001
Aiming distance (m)	−0.283	0.015	−29.080	<0.001
Body size (large)	−0.720	0.498	−1.446	0.148
Body size (medium)	−5.986	0.708	−8.453	<0.001
Camera model (PC900)	−3.285	0.399	−8.226	<0.001
Distance model	7.133	0.671	10.624	<0.001
Speed (metres per second)	−4.923	0.522	−9.435	<0.001
Sun altitude	−2.184	0.878	−2.488	0.013
Sun azimuth	−0.335	0.108	−3.107	0.002
Body size (large) : distance model	0.387	0.740	0.523	0.601
Body size (medium) : distance model	3.678	0.789	4.663	<0.001
Camera model (PC900) : distance model	2.088	0.624	3.347	<0.001

The two camera models exhibited different strengths relative to our experimental design. The PC900 has a taller vertical field of view (32°) than the HP2X (30°) (Reconyx, Inc.) and, as a result, had a higher probability of detecting proxies located 2 m in front of the camera compared to the HP2X (Fig. 3). Marginal effects show that this was particularly important for detecting the medium-sized proxy when the camera lens was ≥116 cm above the ground. In contrast, the HP2X had a higher probability of detecting proxies located >6 m in front of the camera.

Sun position exhibited small, but significant effects on detection probabilities. Detection declined as the sun rotated towards the front of the camera (*p*≤0.013); the mean probability of detection was 0.73 (SD = 0.319) during the morning when the sun was oriented 92 to 124° relative to the camera lens, and 0.69 (SD = 0.329) during the afternoon when the sun was oriented 41 to 88° relative to the camera lens.

## Discussion

We modelled detection as a Bernoulli process, where each trial results in a success (detection) or failure (miss). Ecologists rarely estimate false negatives directly and have developed numerous analytical methods that estimate *r*_*t*_ indirectly using information about the encounter process (*r*_*e*_). Early camera trap studies estimated detection by applying capture-recapture methods to marked individuals (Karanth & Nichols, 1998). Marking is not feasible for many species, leading to the development and application of random encounter (Rowcliffe et al., 2011), spatial count (Chandler & Royle, 2013), distance sampling (Howe et al., 2017), time- and space-to event (Moeller et al., 2018), and random encounter and staying time models (Nakashima et al., 2018; Warbington & Boyce, 2020). These models are needed to make robust inferences about population state variables and can be improved by deploying cameras in such a way that they maximize *r*_*t*_. We provide a simple, adaptable experimental and analytical framework that ecologists, citizen scientists, and others can use to maximize detection probabilities (*r*_*t*_) for a given wildlife species or community.

A camera’s ability to detect an animal is determined by the arrangement of the PIR sensor array relative to the size of that animal’s heat signature (Welbourne et al., 2016). As a result, the detection process arises from the properties of the animal, the camera, and how the camera is deployed. For example, detection necessarily declines with distance because animals’ heat signatures are proportional to their body size and decreases with distance according to the inverse-square law (Papacosta & Linscheid, 2014). Similarly, camera models’ performance can vary under similar ecological conditions (Driessen et al., 2017; Heiniger & Gillespie, 2018; Swann et al., 2004) because their sensitivity is influenced by the physical arrangement of the PIR sensors relative to the animal. Finally, the physical placement of the camera can influence performance by altering the orientation of the sensor array relative to the same heat signature.

Lens height and angle are two parameters that ecologists can easily manipulate in the field. Previous studies suggest that the importance of lens height is mediated by how camera sensors are angled relative to animal movement. For example, cameras deployed 60–90 cm above ground level detected more animals than cameras deployed at 3 m with the sensors oriented parallel to a road (Meek et al., 2016), but similar numbers of animals when cameras were deployed at 3 m with sensors were oriented to cover an entire hiking trail or game trail (Jacobs & Ausband, 2018). Although correlations prevented us from isolating the effects of camera angle and lens height, our regression models show that deploying cameras <90 cm and parallel to the ground (i.e. near 0°) resulted in the highest detection probabilities, consistent with the understanding that detection is highest when cameras point at the centre body mass of target species (Meek et al., 2014, 2016). This relationship presents a challenge where snow accumulation creates time-varying *r*_*t*_ vis-à-vis variation in effective height. Ecologists using camera traps in temperate, boreal, and arctic climates may need to consider seasonal camera protocols (e.g. deploying cameras at different heights) or modelling detection as a spatio-temporal process. Applying our experimental and analytical framework across a range of lens heights and angles using a full-factorial design could help ecologists identify deployment parameters that are less sensitive to time-varying *r*_*t*_.

Although not a focus of our study, we found that detection probabilities were higher when the sun was behind the camera (Table 2). As PIR sensors detect heat emitted from the surface of an object, we suspect that the accumulation of radiant energy throughout the day reduced detection by homogenizing surface temperature of species and background objects (Welbourne et al., 2016). Although the mean probability of detection in our experiment only declined 0.04 from morning to afternoon, our results suggest that unmodeled heterogeneity arising from solar radiation can bias inferences where animal movements vary over time (Frey et al., 2017; Green et al., 2022). Existing camera protocols that recommend aiming camera traps north to avoid sun glare that can impact photo quality (e.g. ABMI, 2021) do not fully account for heterogeneous detection that can arise from solar position. For example, unmodeled heterogeneity arising from solar position may wax and wane across seasons, particularly at high latitudes the range of solar radiation is greatest. We suggest biologists consider solar conditions when designing and analyzing camera trap studies.

There are at least three general frameworks ecologists can use to estimate detection probabilities (*r*_*t*_). Monitoring the same location using multiple cameras (Jacobs & Ausband, 2018; Meek et al., 2016) allows ecologists to estimate relative *r*_*t*_ using realistic conditions but can be inefficient to assess detection across a range of covariate values and may confound *r*_*t*_ with *r*_*e*_ (however see Palencia et al., 2022). Directly assessing false positives from time-lapse images (Hamel et al., 2013) allows ecologists to estimate absolute *r*_*t*_ | *r*_*e*_ using realistic conditions but is similarly inefficient across a range of covariate values. Directly assessing false positives in an experimental framework (Apps & McNutt, 2018, this study) allows ecologists to efficiently estimate absolute *r*_*t*_ | *r*_*e*_ across a range of covariate values at the cost of realistic animal size and movement. Although we applied distance sampling methods in an experimental framework, they can be adapted to estimate absolute *r*_*t*_ in any framework where the distance between the camera (i.e. observer) and animal are measured without bias.

Ecologists can easily extend our experimental and analytical framework; for brevity, we identify four extensions. First, detection errors can also occur when an unidentified animal triggers a camera trap (*r*_*p*_). Identifiability can be limited by camera flash, trigger speed, image quality, weather, lighting, vegetation density, and animal speed, direction, and coat colour relative to background conditions. Conducting experimental trials across a range of these conditions could further help ecologists quantify conditions that impact species identification, test temporally dynamic detection models, and develop protocols that maximize identifiability of target species. Second, as alluded to above, ecologists can apply it to more realistic scenarios. For example, the medium and large proxies in our study had the same body mass and surface area, even though animals emit heat in proportion to their surface area (Martin & Barboza, 2020; Mortola, 2013). Conducting experimental trials where the target closely resembles the study species size and movement—and is allowed to enter and exit the field of view from multiple directions and angles—is needed to achieve ecologically credible estimates (Apps & McNutt, 2018; Becker et al., 2022). Proxies are valuable for exploring mechanisms and trade-offs associated with camera deployments but cannot fully replace target species. Third, our results show that mixing two camera models made by the same manufacturer can have measurable impacts on detection within a given study. Conducting experimental trials on more camera models can help ecologists identify risks (Palencia et al., 2022), and specify analytical models aimed at correcting heterogeneous detection arising from using multiple camera models. Finally, ecologists can estimate detection probabilities by incorporating covariates into the empirical detection functions (Appendix 3). The high number of parameters relative to the data in our experiment resulted in a complex likelihood surface with several local maxima, so we conditioned covariates on the estimated detection function. Reducing the number of parameters in future experiments (e.g. fewer body sizes) or increasing sample sizes could facilitate the development of more robust models where the shape of the distance model varies dynamically based on other covariates.

Our framework does not replace the need to consider detection processes in the analysis of camera trap data (Hofmeester et al., 2019). It can be used to develop field protocols that maximize detection probabilities (*r*_*t*_) for a given wildlife species, or assess potential trade-offs among species, seasons, camera models, and plot layout. Ecologists can also apply our experimental and analytical framework to field data by assessing how many time-lapse images containing an animal or a fresh animal track (trials) resulted in a triggered image (success or failure). Relating these trials to site-specific data about camera model, angle, height, and animal distance can be used to estimate species- and deployment-specific detection probabilities without needing to collect additional information about the encounter process.

## Conclusion

Our study shows how experimental trials can reduce uncertainty associated with detection bias in *r*_*t*_ when designing and analyzing camera trap programs. It may be reasonable to assume near-perfect detection for some species-distance combinations so long as vegetation and topography do not obscure animals (Becker et al., 2022; Keim et al., 2019); however, these scenarios are often limited to specific ecological conditions. Biologists are therefore faced with the choice of censoring data to distances where detection is homogenous and nearly perfect or incorporating species-specific models that account heterogeneous detection probabilities. Conducting trials across a range of conditions relevant to a given study area can help quantify detection zones and thus help biologists optimize camera protocols, account for variation arising from using multiple camera models, and assess trade-offs that arise from interactions among distance, cameras, and body sizes. Ultimately, this information can help users obtain unbiased estimates of animal use, occupancy, and density to support management or conservation activities.

### Supplementary information

ESM 1 Appendix 1. Supplementary figures (PDF 475 kb)

ESM 2 Appendix 2. Horizontal field of view and vertical angle (PDF 110 kb)

ESM 3 Appendix 3. Demonstration code (R 20.3 kb)

## Data Availability

The datasets generated and analyzed during this study are available from the corresponding author on reasonable request.
