# Supplementary material for: A simple framework for maximizing camera trap detections using experimental trials
Source: Environ Monit Assess. 2023 Oct 27;195(11):1381. doi: 10.1007/s10661-023-11945-9 (PMC10611648; doi:10.1007/s10661-023-11945-9)

A simple framework for maximizing camera trap detections using experimental trials

Philip D. DeWitt[1], Amy G. Cocksedge[1]

[1]Ministry of Natural Resources & Forestry, 300 Water Street, Peterborough, Ontario K9J 3C7, Canada

Environmental Monitoring and Assessment

Article DOI: https://doi.org/10.1007/s10661-023-11945-9

Appendix 1. Supplementary figures

Fig. A1-1 Generalized layout of the experimental plot shown from above. We placed stakes (dark grey circles) 2, 4, 6, 8, 10, 12, and 15 m from the front of the camera array (open square) and marked the maximum extent of the plot by placing stakes at 1 m intervals perpendicular to the 15-m stake. Trials were conducted by moving perpendicular to the camera into, across, and out of the cameras' field of view (diagonal, dotted lines) at each of the distances (solid lines)

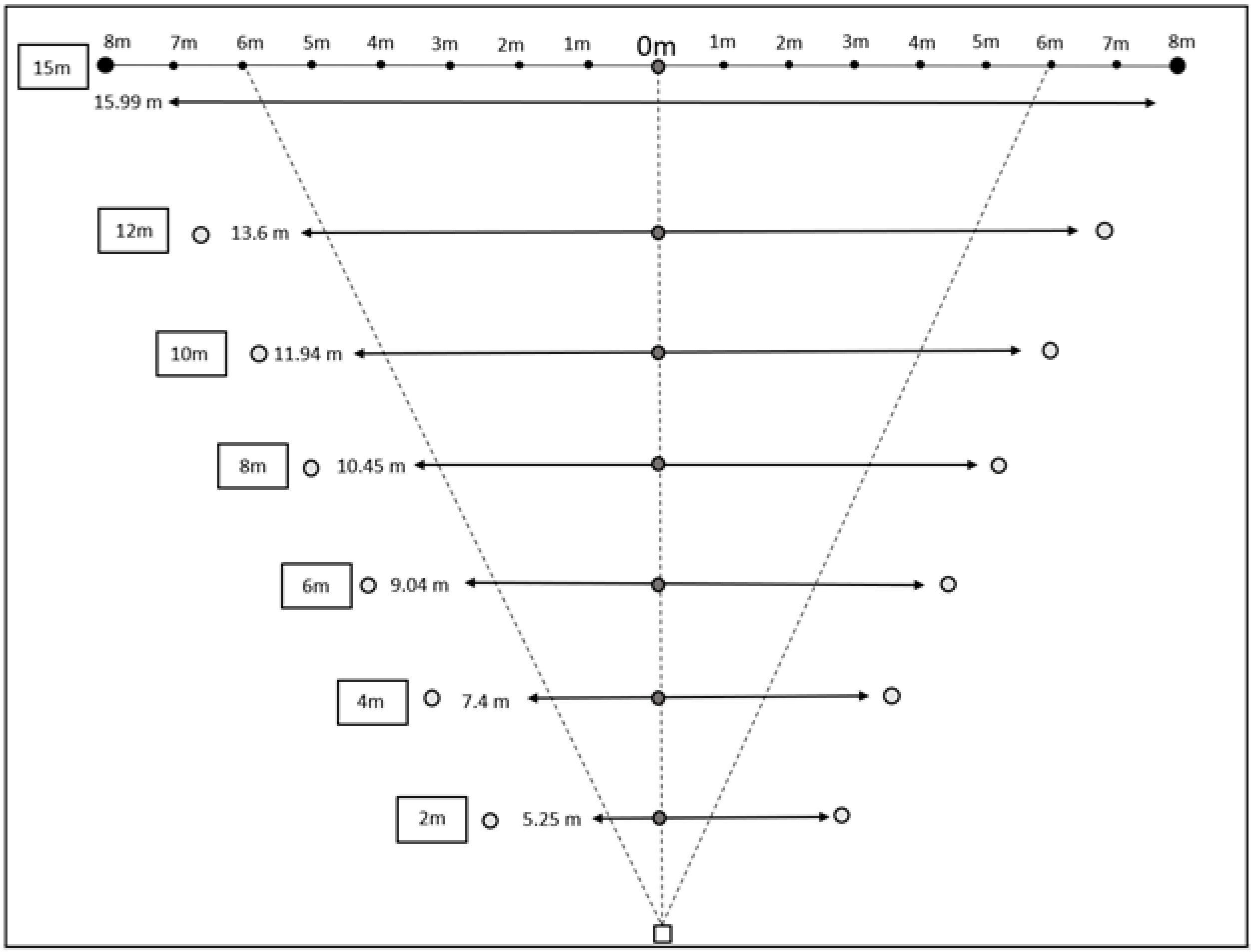

Fig. A1-2 Generalized layout of the experimental plot shown from behind the camera array. A generalized field of view (diagonal, dotted lines) extends from the camera array to the maximum extent of the plot. A yellow and black snow measurement gauge is located in front of the camera array.

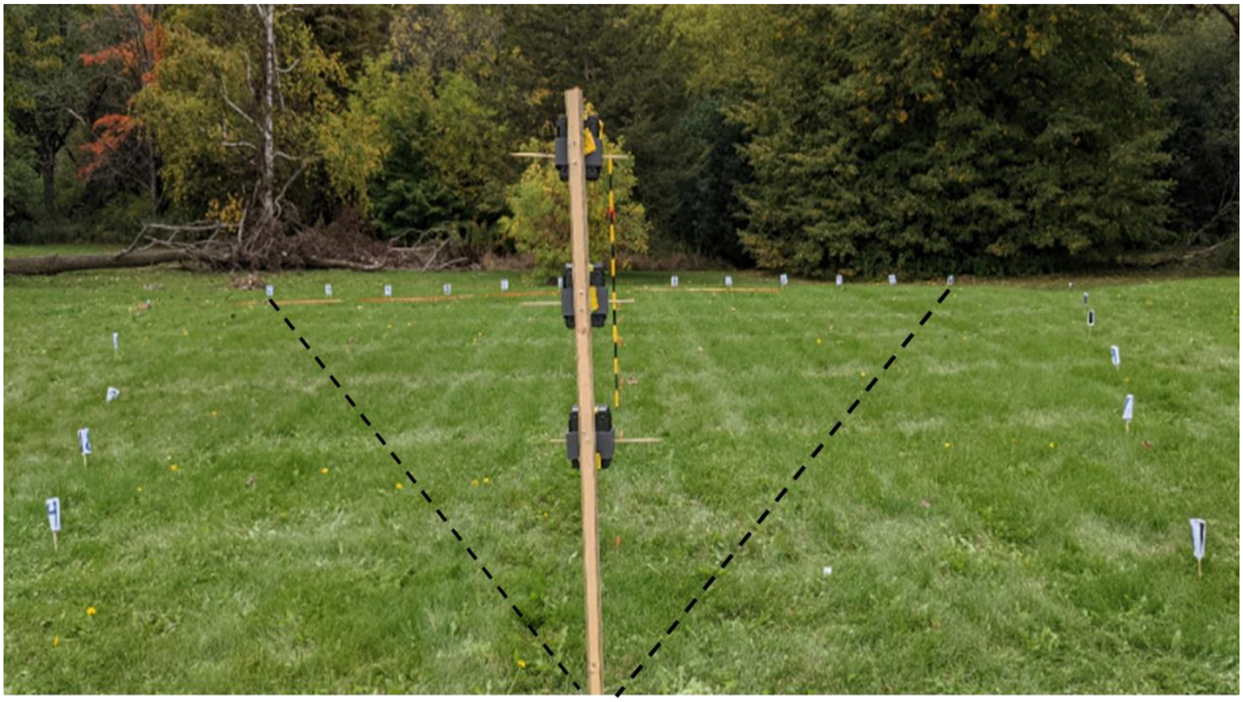

Fig. A1-3 The mean estimate (points) and 95% confidence intervals (bars) of parameters included in both statistical models. Distance refers to the fitted half-normal-logistic mixture model. Body size includes large ungulates (reference category), large, and medium. Camera model includes HP2X (reference category) and PC900. Statistical interactions are denoted with a colon.

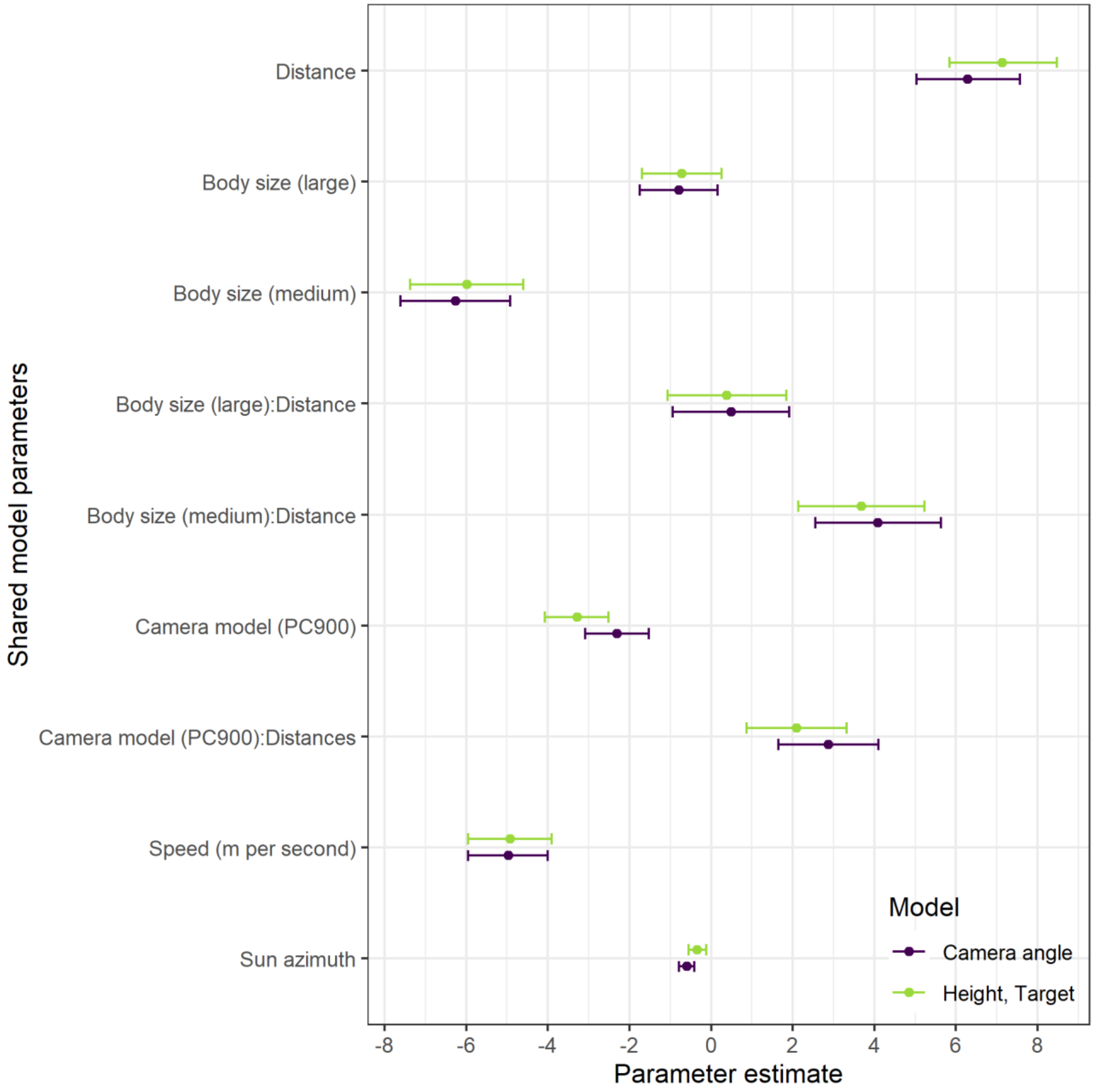

Fig. A1-4 Receiver-operating characteristics curve for the binomial regression model including vertical camera angle. The curve shows the proportion of correctly classified detections (sensitivity) against the proportion of correctly classified misses (specificity), and the area under the curve (AUC). The point along the curve shows the optimal threshold.

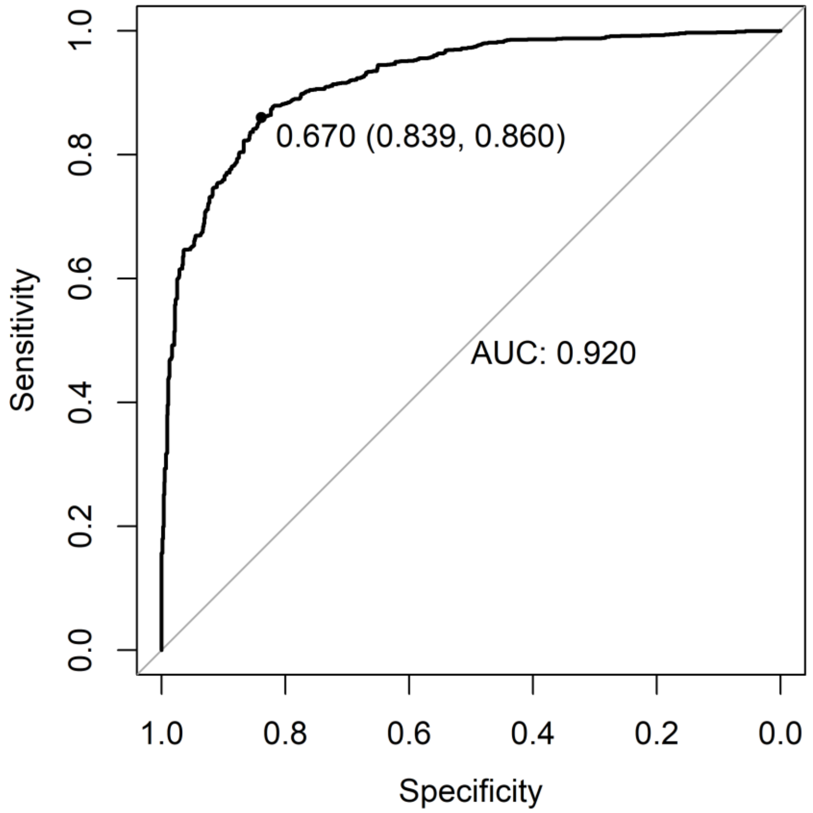

Fig. A1-5 Receiver operating characteristics curve for the binomial regression model including lens height and aiming distance. The curve shows the proportion of correctly classified detections (sensitivity) against the proportion of correctly classified misses (specificity), and the area under the curve (AUC). The point along the curve shows the optimal threshold.

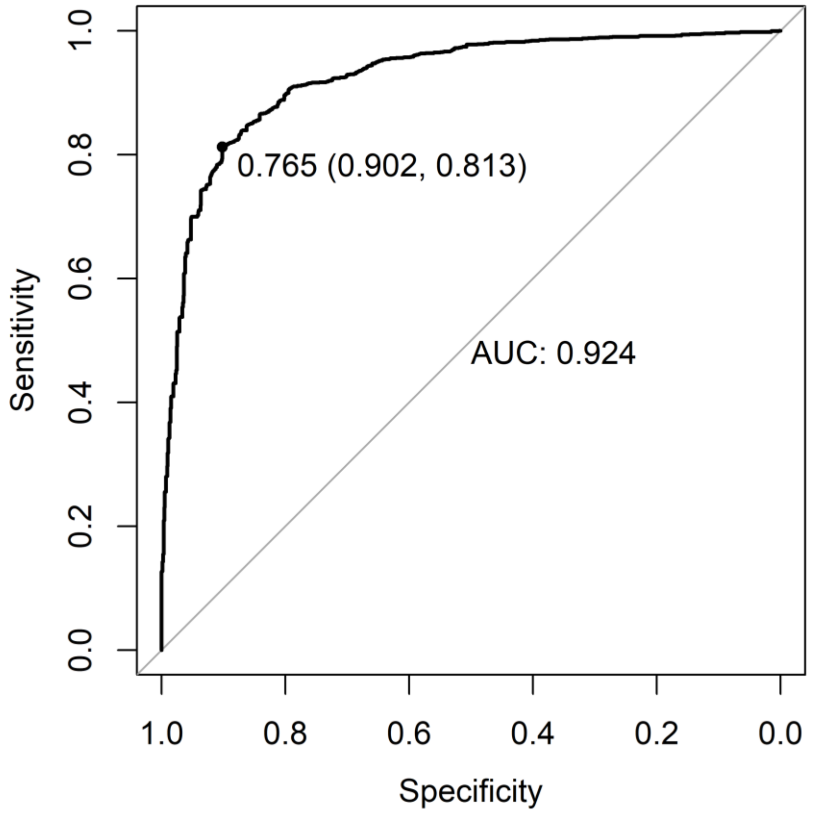

Supplement: Supplementary file 1 — Appendix 1. Supplementary figures (PDF 475 kb) [file 10661_2023_11945_MOESM1_ESM.pdf]
