# Supplementary material for: A simple framework for maximizing camera trap detections using experimental trials
Source: Environ Monit Assess. 2023 Oct 27;195(11):1381. doi: 10.1007/s10661-023-11945-9 (PMC10611648; doi:10.1007/s10661-023-11945-9)
Supplement: Supplementary file 2 — Appendix 2. Horizontal field of view and vertical angle (PDF 110 kb) [file 10661_2023_11945_MOESM2_ESM.pdf]

A simple framework for maximizing camera trap detections using experimental trials

Philip D. DeWitt[1], Amy G. Cocksedge[1]

[1]Ministry of Natural Resources & Forestry, 300 Water Street, Peterborough, Ontario K9J 3C7, Canada

Environmental Monitoring and Assessment

Article DOI: https://doi.org/10.1007/s10661-023-11945-9

Appendix 2. Horizontal field of view and vertical angle

We measured the horizontal field of view, the width of an area seen in an image, at each lens height when cameras were positioned to focus the snow measurement gauge located 5m in front of the camera array. The horizontal field of view was calculated by placing stakes at 1 m intervals perpendicular to each of the distances (2, 4, 6, 8, 10, 12, 15 m) and measuring the maximum width visible at each distance resulting in a balanced dataset of 42 data points. We mapped each location and calculated the half-angle of the field of view for the PC900 ($\bar{x}$ = 21.0, σ_x= 0.76, n = 21) and HP2X ($\bar{x}$ = 19.0, σ_x= 0.75, n = 21) using Pythagoras' theorem and the law of cosines.

We used linear regression to assess the combined effect of distance, lens height, and camera model on the horizontal field of view (Table A2.1). The cameras' fields of view increased an average of 74 cm per metre of distance and that the PC900's field of view was 70 cm wider at any given distance (Table A2.1). In contrast, field of view only increased 0.37 cm per metre of lens height. The effects of distance and camera model arise from the geometry and

shape of camera lenses, however, the reported effects of lens height should be considered preliminary as we did not replicate measurements.

Table A2.1. Parameter estimates and standard errors in the selected linear regression model estimating the width of the field of view 2 to 15 metres in front of a camera. Camera model includes HP2X (the reference category) and PC900.

**Covariates**	**Parameter estimate**	**Standard error**	**Z value**	**Pr(|>t|)**
Intercept	–0.828	0.245	–3.380	0.002
Distance (m)	0.740	0.011	67.678	<0.001
Lens height (m)	0.367	0.188	1.946	0.059
Camera model (PC900)	0.698	0.092	7.558	<0.001

We used a digital level to measure the vertical angle of the camera body to the nearest 0.1 degree when cameras were positioned to focus the snow measurement gauge located 5, 10, or 15 m in front of the camera array. Twenty measurements were collected for every combination of camera model (PC900, HP2X), lens height (86, 116, 146 cm), and snow gauge distance (5, 10, 15 m) resulting in a balanced dataset of 360 data points. We used linear regression to assess the combined effect of aiming distance, lens height, and model on vertical angle (Table A2.2).

The regression model had an adjusted $R^2 = 0.83$, $F_{(5, 354)} = 341.4$, and residuals were normally distributed around the mean. Vertical angles were more pronounced as lens height increased, and lens height was less important as the aiming distance increased. We found that aiming distance was less impactful on the vertical angle of the PC900, likely because it has a taller vertical field of view (32°) than the HP2X (30°)(Reconyx, Inc). The estimated mean and 95% prediction intervals from the model are provided in Table A2.3.

Table A2.2. Parameter estimates and standard errors in the linear regression model estimating the vertical camera angle measured in degrees. Camera models includes HP2X (the reference category) and PC900. Statistical interactions are denoted with a colon.

**Covariates**	**Parameter estimate**	**Standard error**	**t value**	**Pr(|>t|)**
Intercept	14.722	0.863	17.052	<0.001
Aiming distance (m)	–1.142	0.075	–15.197	<0.001
Lens height (m)	–13.347	0.717	–18.620	<0.001
Camera model (PC900)	–1.849	0.674	–2.746	0.006
Aiming distance (m) : lens height (m)	0.677	0.062	10.900	<0.001
Aiming distance (m) : camera model (PC900)	0.085	0.030	2.806	0.005
Lens height (m) : camera model (PC900)	–1.206	0.507	–2.378	0.018

Table A2.3. Estimated mean (lower and upper 95% prediction intervals) vertical angle of the camera body relative to ground surface for two camera models, three aiming distances, and three lens heights. Estimates are shown in degrees and are based on the fitted model.

**Camera model**	**Metres to target**	**86 cm above ground**	**116 cm above ground**	**146 cm above ground**
PC900	5	–2.16 (–4.51, 0.19)	–5.37 (–7.70, –3.04)	–8.58 (–10.93, –6.22)
PC900	10	–4.39 (–6.72, –2.06)	–6.73 (–9.05, –4.418)	–9.06 (–11.39, –6.73)
PC900	15	–6.62 (–8.97, –4.27)	–8.09 (–10.41, –5.76)	–9.55 (–11.90, –7.20)
HP2X	5	0.59 (–1.77, 2.94)	–2.55 (–4.87, –0.22)	–5.68 (–8.03, –3.33)
HP2X	10	–2.36 (–4.69, –0.03)	–4.33 (–6.65, –2.01)	–6.31 (–8.63, –3.98)
HP2X	15	–5.30 (–7.65, –2.95)	–6.12 (–8.45, –3.79)	–6.93 (–9.29, –4.58)
